# Artesunate suppresses Th17 response via inhibiting IRF4-mediated glycolysis and ameliorates Sjog̈ren’s syndrome

**DOI:** 10.1038/s41392-022-01103-x

**Published:** 2022-08-29

**Authors:** Fan Xiao, Ke Rui, Man Han, Liyun Zou, Enyu Huang, Jie Tian, Lijun Zhang, Quan Jiang, Yuzhang Wu, Liwei Lu

**Affiliations:** 1 Department of Pathology and Shenzhen Institute of Research and Innovation, Shenzhen Hospital, The University of Hong Kong; Chongqing International Institute for Immunology, Hong Kong, China; 2 Department of Laboratory Medicine, Affiliated Hospital and Institute of Medical Immunology, Jiangsu University, Zhenjiang, China; 3 Division of Rheumatology, Guang’anmen Hospital, China Academy of Chinese Medical Sciences, Beijing, China; 4 Institute of Immunology, Army Medical University, Chongqing, China

**Keywords:** Translational immunology, Inflammation, Rheumatic diseases

**Dear Editor**,

Primary Sjögren’s syndrome (pSS) is an autoimmune disease characterized by dry eyes and dry mouth caused by glandular inflammation in salivary glands (SG) and lacrimal glands. Currently, pSS patients are suffering from a lack of effective therapies. Many studies have revealed dysregulated immune responses during pSS development, in which Th17 cells are considered as the key driver in disease initiation and perpetuation.^1,2^ Previous studies suggested that artesunate (ART), an important derivative of artemisinin and a first-line antimalarial agent, modulated Th17 and regulatory T (Treg) cells balance in rats with collagen-induced arthritis.^3^

In this study, we first found that ART effectively inhibited both murine and human Th17 proliferation and differentiation in culture (Supplementary Fig. S1a-i). Notably, ART exhibited diverse effects on T cell subsets but most profoundly affected Th17 cells in culture (Supplementary Fig. S2a–d). In mice with induced experimental SS (ESS),^2^ administration of ART markedly improved salvia secretion and significantly reduced serum levels of autoantibodies including anti-SSA IgG during ESS disease progression (Fig. 1a–b). ART treatment dramatically reduced Th17 cells but slightly decreased Th1 cells with no obvious effects on other T cell subsets in ESS mice (Fig. 1c, Supplementary Fig. S3). Histological examination revealed severe SG inflammation and tissue destruction in vehicle-treated ESS mice, but only mild SG inflammation in ART-treated ESS mice (Fig. 1d–e). Importantly, transfer of congenic CD45.1^+^ Th17 cells into IL-17-deficient mice (CD45.2^+^) induced saliva secretion dysfunction, whereas ART treatment significantly improved saliva flow rates in these mice (Supplementary Fig. S4a–b). Notably, ART-treated mice exhibited markedly decreased numbers of CD45.1^+^ donor Th17 cells compared to vehicle-treated counterparts (Supplementary Fig. S4c–d).

**Fig. 1:**
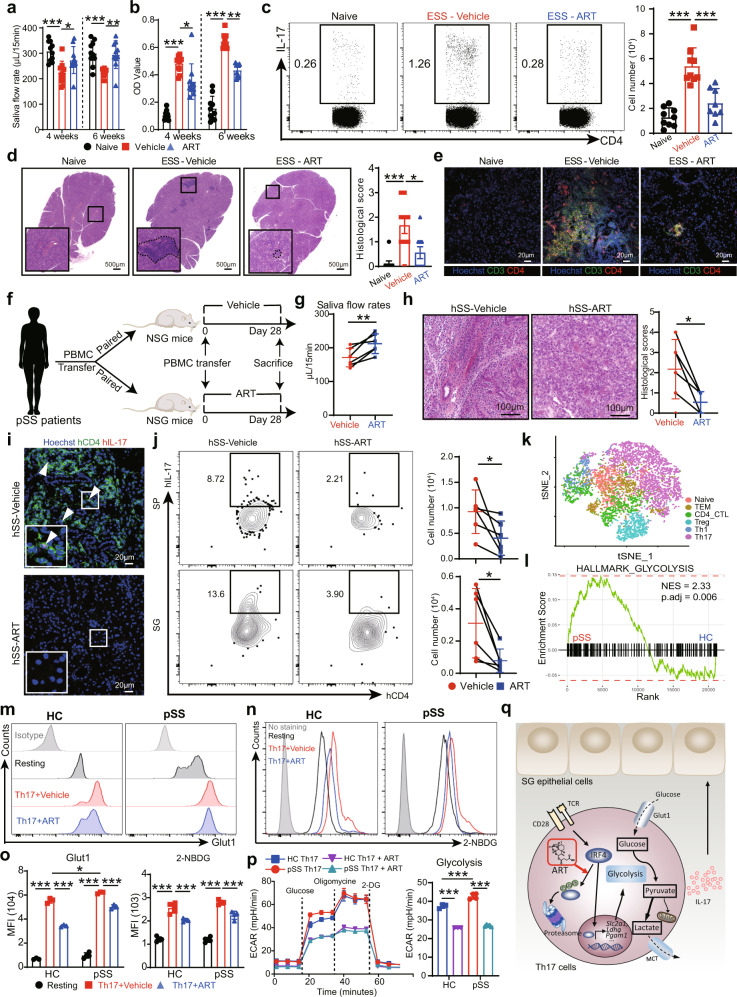
ART suppresses Th17 response via inhibiting glycolysis and ameliorates Sjog̈ren’s syndrome development. **a** ESS mice were induced and treated with vehicle or ART. Saliva flow rates were measured (*n* = 10). **b** The levels of anti-SSA IgG in serum were measured by ELISA (*n* = 10). **c** Representative flow cytometric profiles showing Th17 cells in draining cervical lymph nodes were presented. The numbers of Th17 cells were enumerated (*n* = 8–9). **d** Representative H&E staining images of SG tissues were shown. Histological scores were analyzed (*n* = 9). **e** Representative confocal images showing T cells infiltration in SGs were presented. **f** The schematic diagram shows the generation of humanized SS mice and ART treatment. **g** Saliva flow rates were measured on day 28 after cell transfer (*n* = 6). **h** Representative H&E staining images of SG tissues were shown. Histological scores were analyzed. **i** Representative confocal images showing infiltrating human Th17 cells (arrows) in SG. **j** Human Th17 cells in spleen (SP) and SG were analyzed by flow cytometry (*n* = 6). **k** T-SNE visualization of CD4 T cells from PBMC of healthy controls (HC) and pSS patients. **l** GSEA plots showing an enrichment of glycolytic signatures in Th17 from pSS patients compared with those from HC. **m**–**o** Purified CD4 T cells from healthy control (HC) and pSS patients were cultured under Th17 polarization conditions and treated with vehicle or ART. CD4 T cells without stimulation were collected as resting cells. The expression levels of Glut1 were detected by flow cytometry (**m** and **o**, *n* = 3). The cultured Th17 cells were incubated with 2-NBDG solution for glucose uptake assay. The fluorescence intensities were analyzed by flow cytometry (**n**–**o**, *n* = 4–5). **p** The cultured Th17 cells from HC and pSS patients were treated with vehicle or ART and collected for seahorse glycolysis stress test. ECAR values were monitored with sequential addition of glucose, oligomycin, and 2-deoxy-glucose (2-DG). The ECAR values representing glycolysis were calculated and analyzed (*n* = 3–5). **q** Schematic figure illustrates that ART increases proteasomal degradation of IRF4 and suppresses glycolysis in Th17 cells. **f** Includes modified images from Servier Medical Art (http://www.servier.com). Data were obtained from at least three independent experiments and presented as mean ± SD; one-way ANOVA (**a**–**d, o**–**p**) and paired *t*-test (**g**, **h**, **j**); *P < 0.05; **P < 0.01; ***P < 0.001

Using a humanized SS model in NOD-SCID IL2Rγnull (NSG) mice, we transferred peripheral blood mononuclear cells (PBMC) from pSS patients into NSG mice and found that PBMCs from pSS patients but not healthy donors decreased saliva secretion and caused intensive SG inflammation in recipient NSG mice (Supplementary Fig. S5a–c). To evaluate the effects of ART on human Th17 cells in vivo, PBMC samples from pSS patients were divided for transferring into paired recipient NSG mice, followed with ART or vehicle treatments (Fig. 1f). Compared with vehicle-treated control mice, ART-treated mice exhibited significantly improved saliva flow rates, reduced glandular inflammation and diminished CD4 T cell infiltration in SG (Fig. 1g–i). In particular, ART treatment significantly reduced human Th17 cells in the spleen and SGs of NSG mice (Fig. 1i–j). However, ART exerted no obvious effects on Th1 and Treg cells (Supplementary Fig. S5d).

Based on the single-cell RNA sequencing (scRNA-seq) data of PBMC from healthy control and pSS patients,^4,5^ we identified Th17 population and further detected increased Th17 cells in pSS patients by flow cytometry (Fig. 1k, Supplementary Fig. S6a–b). Gene set enrichment analysis (GSEA) showed highly enriched glycolytic gene signatures in Th17 cells (Supplementary Fig. S6c–d). Th17 cells showed high Glut1 expression and these cells from pSS patients exhibited elevated levels of Glut1 (Supplementary Fig. S6e–f). Notably, Th17 cells from pSS patients displayed several enriched hallmark gene sets associated with glycolysis including *MYC_TARGETS, MTORC1_SIGNALING*, and *GLYCOLYSIS* (Fig. 1l and Supplementary Fig. S6g–h).

During Th17 cell differentiation, Glut1 expression was significantly elevated whereas ART markedly reduced Glut1 expression in both human and murine Th17 cells in culture (Fig. 1m, o and Supplementary Fig. S7a–b). Consistently, ART treatment resulted in a significant reduction of glucose uptake and lactate production (Fig. 1n, o and Supplementary Fig. S7c–d), suggesting that ART may target glycolytic metabolism in Th17 cells.

To determine the effects of ART on Th17 cell metabolism, we monitored extracellular acidification rate (ECAR) and oxygen consumption rate (OCR) values that reflect metabolic status. Although Th17 cells derived from pSS patients showed increased glycolysis levels compared to healthy donors, ART treatment inhibited glycolysis in Th17 cells from both healthy controls and pSS patients (Fig. 1p). ART treatment also decreased glycolysis levels in Th17 cells from normal and ESS mice (Supplementary Fig. S7d–e). To measure the metabolic phenotypes and metabolic potential of Th17 cells, both ECAR and OCR values were examined under resting and stressed conditions. ART decreased stressed ECAR but not OCR metabolic potential (Supplementary Fig. S7f), suggesting that ART may preferentially affect glycolysis in Th17 cells. Moreover, ART treatment exerted marked effects on the glycolytic gene expression profiles and decreased the expression levels of various key glycolytic genes including *Slc2a1, Aldoa* and *Pgam1* (Supplementary Fig. S7g). As expected, inhibition of glycolysis by 2-DG significantly suppressed Th17 differentiation in culture, but ART showed no obvious effects on Th17 cells in the presence of 2-DG (Supplementary Fig. S7h), suggesting that ART targets glycolysis to suppress Th17 cell differentiation. ART decreased glycolysis levels in Th0 and Th1 cells from ESS mice (Supplementary Fig. S8a–d). Compared with Th0 and Th1 cells, Th17 cells showed the highest glycolytic levels (Supplementary Fig. S8e), indicating that Th17 cells are more sensitive to glycolytic inhibition.

IRF4 is a key regulator of T cell differentiation and function. We observed that IRF4-deficient CD4 T cells failed to differentiate into Th17 cells in culture (Supplementary Fig. S9a). IRF4 deficiency resulted in a marked decrease of various key glycolytic genes including *Slc2a1* in CD4 T cells (Supplementary Fig. S9b). In Th17 cells, the levels of Glut1 were associated with IRF4 expression (Supplementary Fig. S9c–d). IRF4-deficient CD4 T cells showed dramatically lower Glut1 expression levels and reduced glucose uptake than those in wild-type (WT) CD4 T cells under Th17 polarization condition although comparable levels of Glut1 expression were detected in resting status (Supplementary Fig. S9e–f). Glycolysis stress tests revealed significantly decreased glycolysis in IRF4-deficient CD4 T cells compared with WT controls. Moreover, ART did not affect glycolysis in IRF4-deficient cells under Th17 differentiation conditions (Supplementary Fig. S9g–h). Together, these results suggest that IRF4 regulates Glut1 expression and controls glycolysis in Th17 cells.

We next examined the effects of ART on IRF4 expression in Th17 cells. ART-treated humanized SS mice exhibited significantly reduced expression levels of IRF4 (Supplementary Fig. S10a). In culture, ART treatment markedly reduced IRF4 levels in both human and murine Th17 cells (Supplementary Fig. S10b–c). Interestingly, ART upregulated the mRNA levels of *Irf4* (Supplementary Fig. S10d), suggesting that ART may exert post-translational effects on IRF4 during Th17 cell differentiation. Notably, we found that ART treatment significantly decreased protein stability and reduced the half-life time of IRF4 but not IRF5 in polarized Th17 cells (Supplementary Fig. S10e–g). Bortezomib, a proteasome inhibitor, increased IRF4 levels while an autophagy inhibitor bafilomycin showed no detectable effects in Th17 cells with ART treatments (Supplementary Fig. S10h–i). Moreover, ART significantly increased the levels of linkage-specific K48 ubiquitin that mainly target proteins for proteasomal degradation in IRF4^+^ Th17 cells (Supplementary Fig. S10j). Upon enhanced CD3 stimulation, T cells showed increased IRF4 expression levels, which partially rescued ART-mediated suppression of Th17 cells in culture (Supplementary Fig. S10k–l). Collectively, these data demonstrate that ART suppresses Th17 glycolysis via increasing proteasomal degradation of IRF4 (Fig. 1q).

In sum, our findings show that ART effectively suppresses disease development in ESS and humanized SS mice via inhibiting Th17 responses. Moreover, we have identified a novel metabolic modulatory function of ART in suppressing Th17 glycolysis through promoting proteasomal degradation of IRF4. Taken together, these findings suggest that ART may serve as a promising therapeutic candidate for treating pSS patients.

## Supplementary information

supplementary material

## Data Availability

All data generated or analyzed in this study are included in this paper and the supplementary material files. The scRNA-seq data are available from Gene Expression Omnibus (GEO) database (GSE157278).^4^ Any data associated with this study are available from the corresponding author upon reasonable request.
