# Supplementary material for: Artesunate suppresses Th17 response via inhibiting IRF4-mediated glycolysis and ameliorates Sjog̈ren’s syndrome
Source: Signal Transduct Target Ther. 2022 Aug 29;7:274. doi: 10.1038/s41392-022-01103-x (PMC9420730; doi:10.1038/s41392-022-01103-x)
Supplement: Supplementary file 1 — supplementary material [file 41392_2022_1103_MOESM1_ESM.docx]

**Supplemental Materials for**

**Artesunate suppresses Th17 response via inhibiting IRF4-mediated glycolysis and ameliorates Sjog̈ren’s syndrome**

Fan Xiao^1^, Ke Rui^2^, Man Han^3^, Liyun Zou^4^, Enyu Huang^1,5^, Jie Tian^6^, Lijun Zhang^7^, Quan Jiang^3^, Yuzhang Wu^4 *^ and Liwei Lu^1,5,7 *^

*Correspondence to:

Liwei Lu (liweilu@hku.hk) and Yuzhang Wu (wuyuzhang@tmmu.edu.cn)

**This supplementary file includes:**

Materials and Methods

Supplementary Figures S1~S10

Supplementary Table S1

Supplementary References

# Materials and methods

## Mice

Female C57BL/6, NOD-SCID IL2Rγnull (NSG), B6.SJL-Ptprca Pepcb/BoyJ (CD45.1^+^) mice were purchased from Jackson Laboratory (Bar Harbor, ME, USA). IRF4-deﬁcient (IRF4^−/−^) mice were kindly provided by Prof. Tak Wah Mak at University of Toronto, Canada. IL-17-deficient mice on a C57BL/6 background were kindly provided by Dr Yoichiro Iwakura at The University of Tokyo, Japan. All mice were housed with ad libitum access to food and water in the Laboratory Animal Unit at The University of Hong Kong. All animal experiments were approved by the Committee on the Use of Live Animals in Teaching and Research at The University of Hong Kong.

## ESS and humanized SS mice

Experimental Sjögren’s syndrome (ESS) induction was performed as previously described.^1,2^ Brieﬂy, the mice were immunized with salivary glands (SG)-derived protein extracts emulsified in Freund’s complete adjuvant (BD Biosciences, San Jose, CA, USA) on day 0 and boosted with the SG proteins in Freund’s incomplete adjuvant (BD Biosciences, San Jose, CA, USA) on day 14 via subcutaneous injection at multiple sites.

Humanized SS mice were constructed as previously described.^3^ Peripheral blood mononuclear cell (PBMC) from pSS patients were transferred into NSG mice. Each PBMC sample from the same donor was transferred into paired recipient NSG mice with equal cell numbers (2.5*10^6 cells/mouse). The collection of PBMC from pSS patients was approved by The University of Hong Kong–Shenzhen Hospital Institutional Review Board (2021-220).

## Artesunate treatment

Artesunate (ART) (Shanghai Aladdin Biochemical Technology Co., Shanghai, China) was dissolved in DMSO and further diluted in PBS for treatments. The ESS mice were treated by ART through oral gavage every day for 7 weeks from the day of first immunization at a dosage of 50 mg/kg body weight. The humanized SS mice were treated with ART (50 mg/kg body weight) or vehicle every day for 4 weeks from the day of cell transfer.

## Saliva flow rate measurement

Saliva ﬂow rates were measured as previously described.^1,4^ Brieﬂy, anesthetized mice were intraperitoneally injected with pilocarpine (Sigma-Aldrich, St. Louis, MO, USA) at a dose of 5 mg/kg body weight. Saliva was immediately collected by a pipet for 15 min at room temperature.

## Histological analysis

Paraffin sections and frozen sections of SGs were prepared by the histology laboratory at the Department of Pathology, The University of Hong Kong. H&E staining was performed using paraffin sections. A widely accepted scoring system based on inﬁltrated lymphocytic foci was adopted to evaluate the severity of the tissue inflammation and damage in ESS mice.^5^ A lymphocytic focus represents a cluster with over 50 lymphocytes. The focus score (FS) was classiﬁed as: FS= 0: no lymphocytic inﬁltration; FS= 1: <1 lymphocytic focus per 4 mm^2^; FS= 2: <2 lymphocytic foci per 4 mm^2^ ; FS= 3: two or more lymphocytic foci per 4 mm ^2^. SG inflammation and tissue damage were scored 0–4 in humanized SS mice ^3^: 0, no inflammatory cells observed (normal); 1, minimal inflammation observed with few inflammatory cells present in the connective tissue between acini and occasional cytoplasmic vacuolation of acinar epithelial cells; 2, mild inflammation characterized by scattered, small clusters of cells in the connective tissue and between acini with nuclear fragmentation of some acinar epithelial cells; 3, moderate inflammation consisting of substantial inflammatory cell presence with larger, coalescing clusters in the connective tissue with a widespread reduction in acinar and duct size; 4, marked inflammation defined by inflammatory cells covering most of the organ and an essential absence of the acinar epithelium.

## Flow cytometry

Flow cytometric analysis was performed as previously described.^6^ Briefly, single-cell suspensions were prepared. The cells were stained with Zombie Aqua solution (BioLegend, San Diego, CA, USA) in PBS for 15 min, followed by incubation with Fc blocker (anti-CD16/32, BioLegend) for another 15 min. After washing with PBS, the cells were stained with fluorochrome-conjugated antibodies against surface antigens for 30 min. To detect IRF4, cells were permeabilized and stained with anti-IRF4 antibodies using eBioscience™ Foxp3 / Transcription Factor Staining Buffer Set (Thermo Fisher Scientiﬁc, Waltham, MA, USA) following the manufacturer’s instructions. To detect cytokines, cells were stimulated with phorbol myristate acetate (50 ng ml^-1^, Sigma-Aldrich), ionomycine (1µg ml^-1^, Sigma-Aldrich) and monensin (2 µM, Biolegend) for 5 hours. After the surface staining, intracellular staining was performed using Cytoﬁx/Cytoperm kit (BD Biosciences) following the manufacturer’s instructions. To detect Glut1, the cells were fixed, permeabilized and stained with primary antibody (anti-Glut1) for 30 min. The cells were then incubated with a secondary antibody for another 30 min. Samples were analyzed using BD LSRFortessa (BD Biosciences). The acquired data were analyzed with FlowJo software (TreeStar). Doublets, debris, and dead cells were excluded during analysis. The following antibodies were used: anti-IL-17 (clone TC11-18H10.1, BioLegend), anti-interferon-γ (anti-IFN-γ, clone XMG1.2, BioLegend), anti-IL-4 (clone 11B11, BioLegend), anti-IL-2 (clone JES6-5H4, BioLegend), anti-Foxp3 (clone 150D, BioLegend), anti-CD4 (clone GK1.5, BioLegend), anti-Glut1(clone SA0377, Thermo Fisher Scientiﬁc), anti-IRF4 (clone IRF4.3E4, BioLegend), anti-IRF5 (clone W16007B, BioLegend), anti-CD45.1 (clone A20, BioLegend), Donkey anti-rabbit IgG (Poly4064, BioLegend), anti-hCD45 (clone 2D1, BioLegend), anti-hCD3 (clone HIT3a, BioLegend), anti-hCD4 (clone OKT4, BioLegend), anti-hIL-17 (clone BL168, BioLegend), anti-hIFN-γ (clone 4S.B3, BioLegend), anti-Ubiquitin (linkage-specific K48, clone EP8589, Abcam, Cambridge, United Kingdom).

## Immunofluorescence staining and confocal microscopy

The frozen sections were ﬁxed with 4% PFA for 15 min at room temperature and washed with PBS three times. The slides were then blocked with 4% BSA for 30 min at room temperature and incubated with fluorochrome-conjugated antibodies at 4 °C overnight. For detection of hIL-17, slides were permeabilized with 0.25% Triton X-100 for 15 min before blocking. Nuclei were counterstained with Hoechst 33258 (Thermo Fisher Scientiﬁc). The samples were observed under LSM780/LSM800 confocal microscopes (Zeiss, Oberkochen, Germany). The following antibodies were used: anti-CD3 (clone 17A2, BioLegend), anti-CD4 (clone GK1.5, BioLegend), anti-hCD4 (clone OKT4, BioLegend), anti-hIL-17 (clone BL168, BioLegend).

## Cell culture

Murine and human Th17 cell differentiation was performed as previously described ^6^. Briefly, Naive CD4^+^ T cells from the spleen and cervical lymph nodes of WT and Irf4^−/−^ C57BL/6 mice were puriﬁed by a Naive CD4 T Cell Isolation Kit (Miltenyi Biotec, Bergisch Gladbach, Germany). The purified naïve T cells were seeded into anti-CD3 and anti-CD28 pre-coated culture plates. For Th17 differentiation, the cells were stimulated with recombinant IL-23 (20 ng/mL, R&D Systems, Minneapolis, MN, USA), IL-6 (25 ng/mL, R&D Systems) and TGF-β (3 ng/mL, R&D Systems) plus anti-IFNg/anti-IL-4 (5 ug/mL, Biolegend) in the absence or presence of ART at different concentrations. In some experiments, the T cells were cultured under Th17 polarization condition with plate-coated anti-CD3 at different concentrations. For Th1 cell culture, T cells were stimulated with IL-12 (20 ng/mL, R&D Systems) plus anti-IL-4 (5 ug/mL, Biolegend). For Th0 cell culture, T cells were stimulated with anti-IFNg/anti-IL-4 (5 ug/mL, Biolegend). For Th2 cell culture, T cells were stimulated with IL-4 (20 ng/mL, peprotech) and anti-IFNg (5 ug/mL, Biolegend). For regulatory T cell culture, T cells were stimulated with IL-2 (100 U/mL) and TGF-β (5 ng/mL, R&D Systems).

Human PBMCs were isolated from buffy coat preparations of blood from healthy donors collected by the Hong Kong Red Cross with written consent. All experiments using human peripheral mononuclear cells of healthy donors were approved by Institutional Review Board of the University of Hong Kong/Hospital Authority Hong Kong West Cluster (HKU/HA HKW IRB). Purified CD4 T cells from PBMC of healthy donors were cultured under Th17 polarization conditions (20 ng/mL recombinant human IL-6, 20 ng/mL recombinant human IL-23, 3 ng/mL recombinant human TGF-β) plus anti-IFNg/anti-IL-4 with or without ART treatments in anti-CD3 and anti-CD28 pre-coated culture plates.

For proliferation assay, the purified CD4 T cells were labeled with 2 μΜ CFSE (Sigma-Aldrich) in a 37 °C water bath for 10 min before cell culture. CFSE ﬂuorescence intensity was measured after culture by ﬂow cytometry.

To determine the half-life time of IRF4 and IRF5, the polarized Th17 cells were collected and treated with vehicle or ART (2µM) in the presence of cycloheximide (100 µg/mL, Abcam) for various time points. IRF4 levels were examined by flow cytometry analysis. The half-life time was determined based on the kinetic curves.

## Adoptive transfer of Th17 cells

Naive CD4 T cells were purified from BoyJ mice (CD45.1+) and cultured under Th17 polarization condition for 3 days. The Th17 cells (3*10^6 cells/mouse) were then intravenously transferred into IL-17 deficient mice (CD45.2+) on day 4 post-immunization for ESS induction. The mice received daily oral gavage of vehicle or ART. On day 14 post-ﬁrst immunization, the mice received a boost immunization. Saliva flow rates were measured and flow cytometric analysis were performed 3 weeks after first immunization.

## Enzyme-linked immunosorbent assay (ELISA)

IL-17 concentrations in culture supernatant and serum were measured using ELISA MAX™ Deluxe Set Mouse IL-17A kit (BioLegend) and IL-17A Human ELISA Kit (Thermo Fisher Scientiﬁc), following the manufacturer’s instructions. The levels of anti-SSA autoantibodies in serum were measured by ELISA as previously described ^6^. The 96-well MaxiSorp plates were coated with SSA peptides (SBS Genetec Co., Ltd., Beijing, China) at 4 °C overnight. The plates were washed and blocked with 0.5% gelatin and 0.5% bovine serum albumin. Serum samples were diluted (1:100), added into plates, and incubated for 2 h at room temperature. Then the plates were washed and incubated with biotin-conjugated anti-mouse IgG (1:1000, BioLegend) for 1 hour. After washing, the plates were then incubated with HRP Streptavidin (1:1000, BioLegend) for 30 min. The plates were completely washed, incubated with freshly prepared TMB substrate (BioLegend) for 10 min. The reaction was stopped by adding 3 M H2SO4 solution. Absorbance at 450 nm was measured using a Sunrise microplate reader (Tecan, Männedorf, Switzerland) within 15 min.

## scRNA-seq analysis

The single-cell RNA sequencing (scRNA-seq) data of PBMC samples from 5 healthy donors and 5 pSS patients were obtained from the GEO database (accession number GSE157278).^7^ The Seurat package was used for quality control, dimensionality reduction by principal component analysis (PCA), and t-Distributed Stochastic Neighbor Embedding (t-SNE) visualization. The quality control standards were set as: (1) genes detected in less than 3 cells were excluded; (2) cells with less than 50 total detected genes were excluded; and (3) cells with over 5% of mitochondria-expressed genes were excluded. CD4 T cells and Th17 cells were identified after unsupervised clustering. Gene set enrichment analysis (GSEA) analysis (<https://www.gsea-msigdb.org/gsea/index.jsp>, category H) was performed to analyze the enrichment of hallmark pathways in Th17 cells and non-Th17 cells.

## Glucose uptake assay

The human CD4 T cells, wild-type (WT) and IRF4 deficient CD4 T cells were cultured under Th17 polarization conditions with or without ART treatments and harvested for glucose uptake assay. The cells were stained with Zombie Aqua solution for 10 min. The cells were washed with PBS and incubated with 2-NBDG solution (100 μg/mL, Thermo Fisher Scientiﬁc) in a cell culture incubator for 20 min. The cells were then washed with cold PBS and subjected to flow cytometric analysis immediately. The FITC channel intensities were recorded and analyzed.

## Metabolic assay

The extracellular acidification rate (ECAR) and oxygen consumption rate (OCR) were determined by XFe96 Extracellular Flux Analyzer (Agilent Technologies). Isolated CD4 T cells were cultured under Th0, Th1 and Th17 polarization conditions with or without ART treatments for 48 hours. Agilent Seahorse XF Glycolysis Stress Test Kit and Cell Energy Phenotype Test Kit (Agilent Technologies) were used to determine glycolysis and energy phenotype of the Th17 cells following the manufacturers’ instructions. The cells were counted, washed with Seahorse XF base medium with required supplements, and seeded into poly-lysine pre-coated XFe96 microplate (1*10^5 cells/well for human T cells, 5*10^5 cells/well for mouse T cells,). For glycolysis stress test, ECAR values were monitored with the sequential addition of 10 mM glucose, 1 μM oligomycin, and 50 mM 2-deoxy-glucose. For the energy phenotype test, ECAR and OCR values were monitored before and after addition of oligomycin (1 μM) plus FCCP (1 μM). The data were analyzed using Seahorse Wave 2.6 software package (Agilent Technologies).

## Quantitative PCR analysis

Quantitative PCR (Q-PCR) analysis was performed as previously described.^1^ The mRNA was extracted using ReliaPrep™ RNA Cell Miniprep System (Promega, Madison, WI, USA) and cDNA was prepared by reverse transcription PCR using PrimeScript™ RT Master Mix kit (Takara, Kyoto, Japan). Q-PCR was performed using TB Green Premix Ex Taq II kit (Takara) following the manufacturer’s instructions. The relative gene expression levels were determined using the 2^ΔΔCt^ method with normalization to 18S rRNA. Primers were listed in Supplementary table 1.

## Statistics

Results were represented as mean ± standard deviation (SD). Student’s *t*-test (paired and unpaired), one-way ANOVA and two-way ANOVA were used as appropriate. *P*-values < 0.05 were considered statistically signiﬁcant.


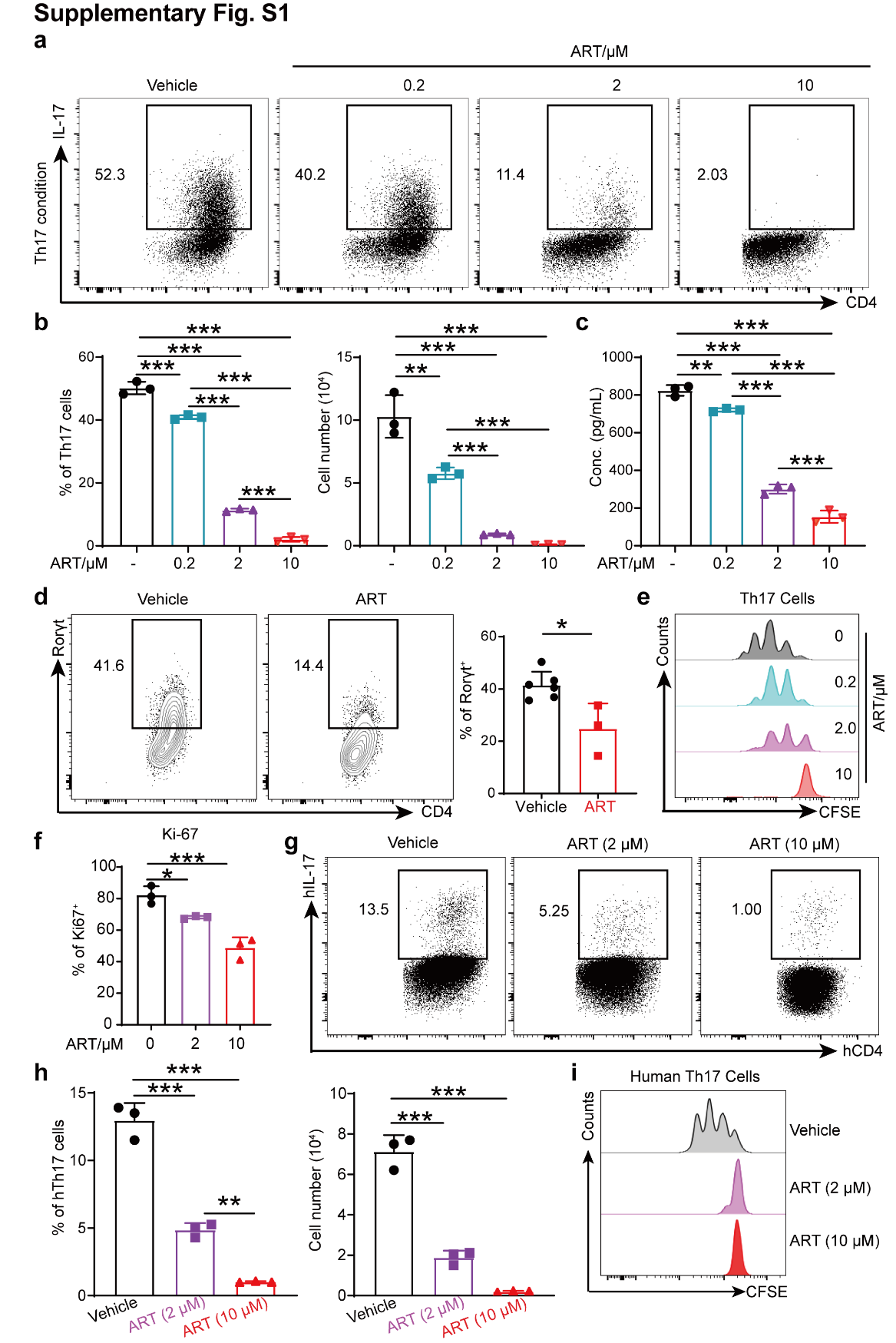


Supplementary Fig S1. ART suppresses Th17 cell generation and proliferation in culture. **a**, Purified murine CD4 T cells were cultured under Th17 polarization conditions and treated with ART at indicated concentrations for 72 hours. CD4^+^IL-17^+^ Th17 cells were detected by flow cytometry. Data are representative of 3 independent experiments. **b**, The frequencies and numbers of Th17 cells were enumerated (n=3). **c**, The concentrations of IL-17 in culture supernatant were measured by ELISA (n=3). **d**, The expression of RORγt in Th17 cells treated with vehicle or ART (2 μM) were examined by flow cytometry (n=3~6). **e**, The CFSE-labelled CD4 T cells were cultured under Th17 polarization conditions and treated with ART. The fluorescence intensities of CFSE were detected by flow cytometry. **f**, The frequencies of Ki67^+^ CD4 T cells were analyzed (n=3). **g**, Purified human CD4 T cells were cultured under Th17 differentiation conditions and treated with ART. The polarized Th17 cells were examined by flow cytometry. **h**, The frequencies and numbers of Th17 cells were determined (n=3). **i**, The fluorescence intensities of CFSE were detected by flow cytometry. Data were obtained from at least three independent experiments and presented as mean ± SD; unpaired t-test (**d**) and one-way ANOVA (**b-c, f, h**); *P < 0.05; **P < 0.01; ***P < 0.001.


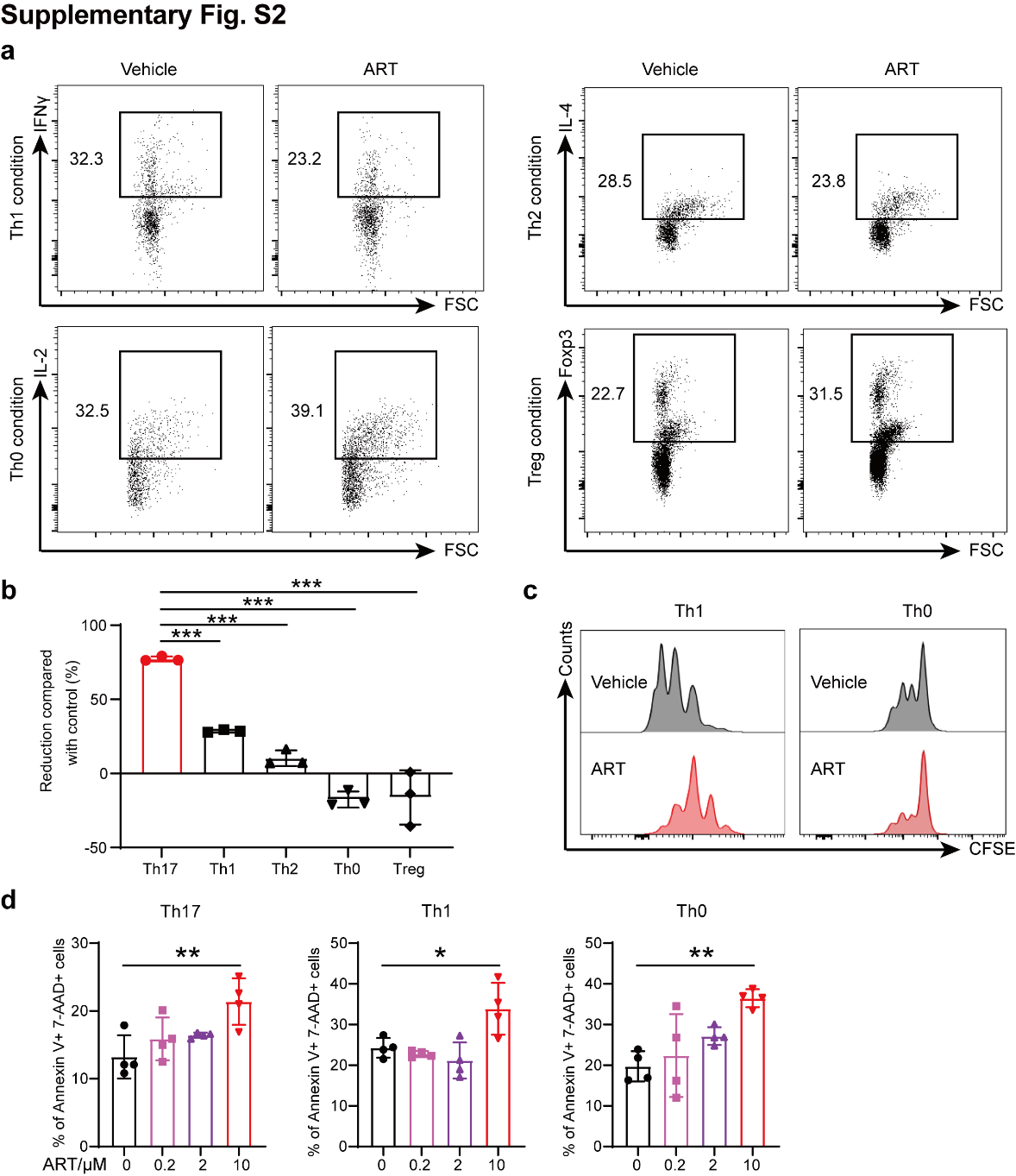


Supplementary Fig S2. The effects of ART on T cell subsets. **a**, Purified naïve CD4 T cells were cultured under indicated polarization conditions and treated with ART (2 μM). IFNγ^+^ Th1 cells, IL-4^+^ Th2 cells, IL-2^+^ Th0 cells and Foxp3^+^ Treg cells were examined by flow cytometry. **b**, The percentages of T cell reduction of ART-treated groups compared with relative controls were analyzed (n=3). **c**, CFSE-labeled cells were cultured under Th0 and Th1 conditions and treated with ART. Cell proliferation were detected by flow cytometry. **d**, Purified CD4 T cells were cultured under indicated polarization conditions and treated with ART at different concentrations. The frequencies of apoptotic cells were analyzed (n=4). Data were obtained from at least three independent experiments and presented as mean ± SD; One-way ANOVA; *P < 0.05; **P < 0.01; ***P < 0.001.


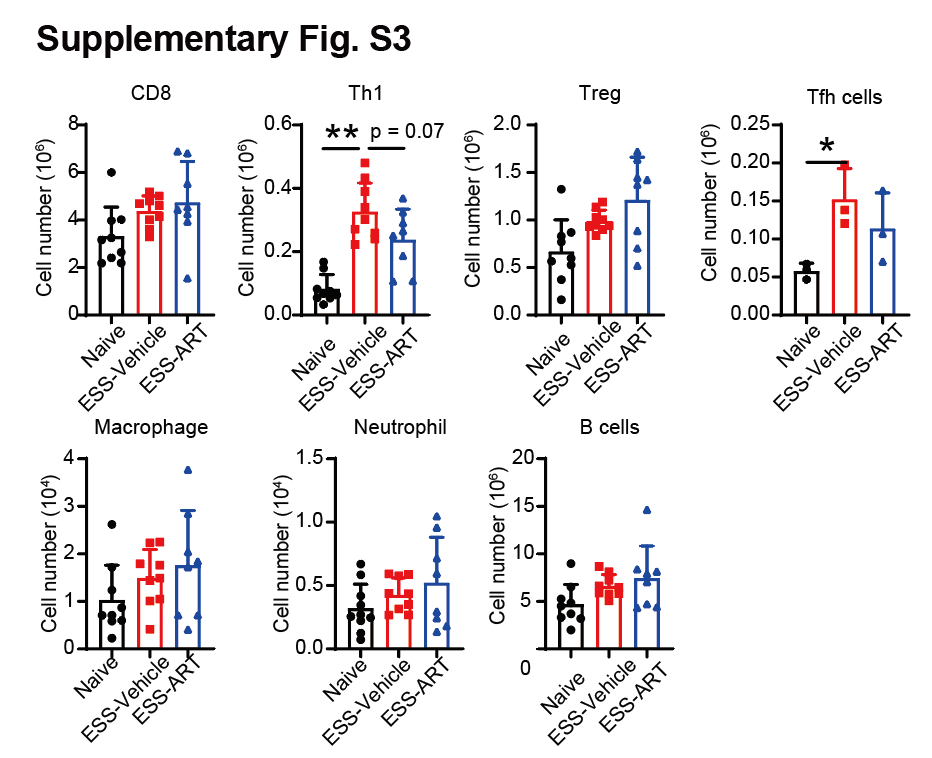


Supplementary Fig S3. The effects of ART on different immune cell types in ESS mice. ESS mice were treated with vehicle or ART. The indicated immune cell populations in draining cervical lymph nodes were analyzed by flow cytometry (n=3~10). One-way ANOVA; *P < 0.05; **P < 0.01; ***P < 0.001.


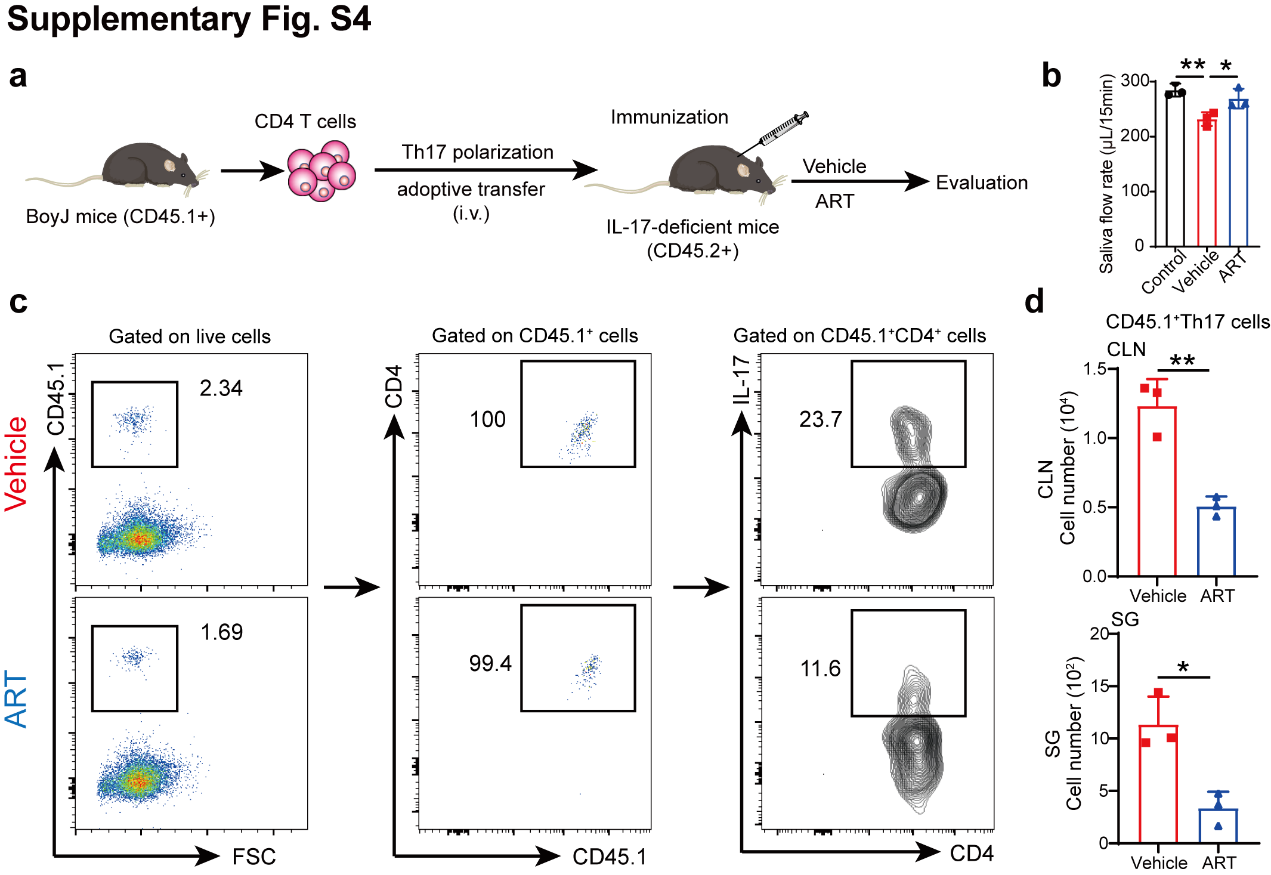


Supplementary Fig. S4. ART suppressed Th17-mediated ESS development. **a**, The schematic diagram showing the experimental procedure. Isolated naïve CD4 T cells from BoyJ mice (CD45.1^+^) were polarized into Th17 cells, which were adoptively transferred into immunized IL-17-deficient mice (CD45.2^+^) for ESS induction, followed by vehicle or ART treatments (n=3). **b**, Saliva flow rates were measured (n=3). Immunized IL-17-deficient mice without cell transfer served as the control group. **c**, Flow cytometric profiles showing donor CD45.1^+^ Th17 cells in salivary glands were presented. **d**, The numbers of donor CD45.1^+^ Th17 cells in cervical lymph nodes (CLN) and salivary gland (SG) were analyzed (n=3). Fig.S4a includes modified images from Servier Medical Art (http://www.servier.com). one-way ANOVA (**b**) and unpaired t-test (**d**); *P < 0.05; **P < 0.01; ***P < 0.001.

_
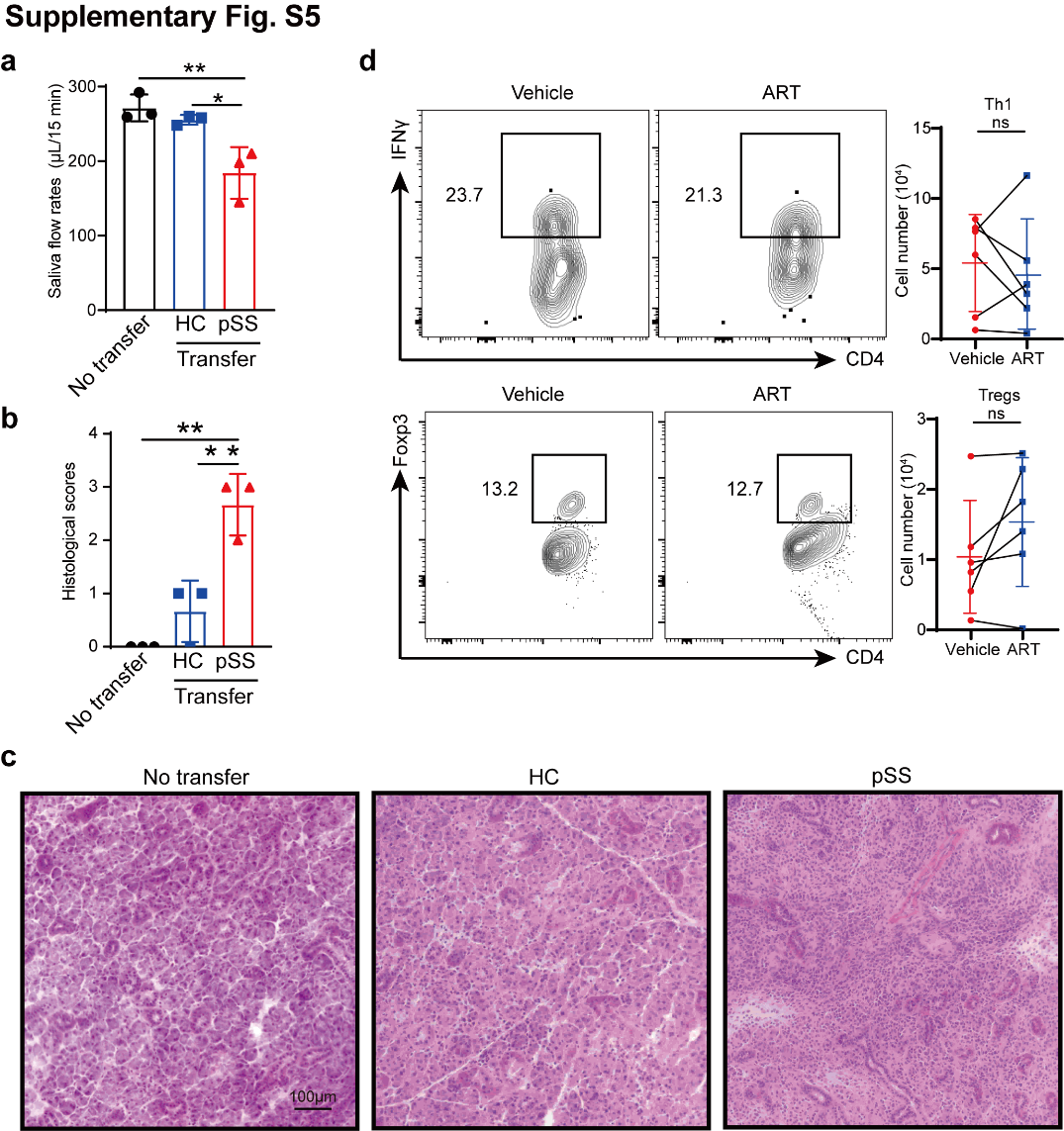
_

Supplementary Fig S5. ART did not affect Th1 and Treg cells in humanized SS mice. **a**, PBMC from healthy control (HC) and pSS patients were transferred into NSG mice. Saliva flow rates were measured at day 28 post-transfer (n=3). **b**, Histological scores were analyzed. **c**, Representative H&E staining images of SGs were shown. **d**, PBMC from pSS patients were transferred into NSG mice and treated with ART or vehicle. Human Th1 cells and Treg cells in spleens were examined by flow cytometry (n=6). One-way ANOVA (**a-b**) and paired t-test (**d**); ns, not significant; *P < 0.05; **P < 0.01; ***P < 0.001.


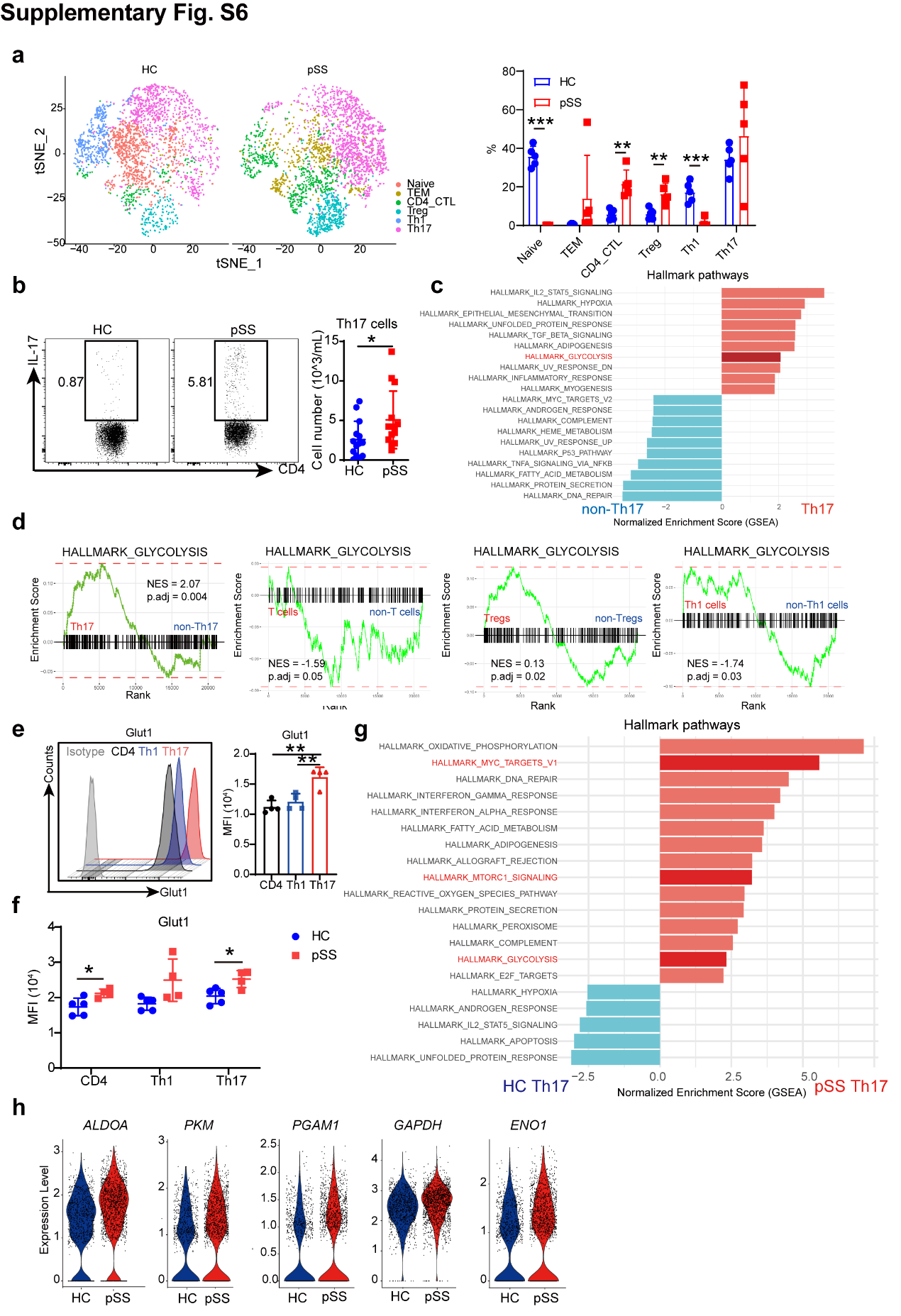


**Supplementary Fig S6.** Enriched glycolytic signatures in Th17 cells of pSS patients. **a**, T-SNE visualization of CD4 T cells from PBMC of healthy controls (HC) and pSS patients, respectively. The frequencies were analyzed (n=5). **b**, Flow cytometric analysis of Th17 cells in PBMC of HC and pSS patients (n=14). **c**, GSEA analysis on the gene expression profiles of Th17 cells and non-Th17 cells from PBMC of HC and pSS patients were performed. **d**, GSEA plots showing the enrichment of glycolytic genes in Th17 cells, T cells, Th1 cells and Treg cells. **e**, The expression levels of Glut1 in total CD4 T (CD4^+^), Th1 (CD4^+^ IFNγ^+^) and Th17 cells (CD4^+^IL-17^+)^ from draining cervical lymph nodes of ESS mice were analyzed by flow cytometry (n=4). **f**, The expression of Glut1 in CD4 T, Th1, and Th17 cells from HC and pSS patients were detected by flow cytometry (n=4-5). **g**, GSEA analysis on the gene expression profiles of Th17 cells from HC and Th17 cells from pSS patients. **h**, Violin plots showing the expression levels of key glycolytic genes in Th17 cells from HC and pSS patients. Data were obtained from at least three independent experiments and presented as mean ± SD, unpaired t-test (**a-b, f**) and one-way ANOVA (**e**), *P < 0.05; **P < 0.01; ***P < 0.001.


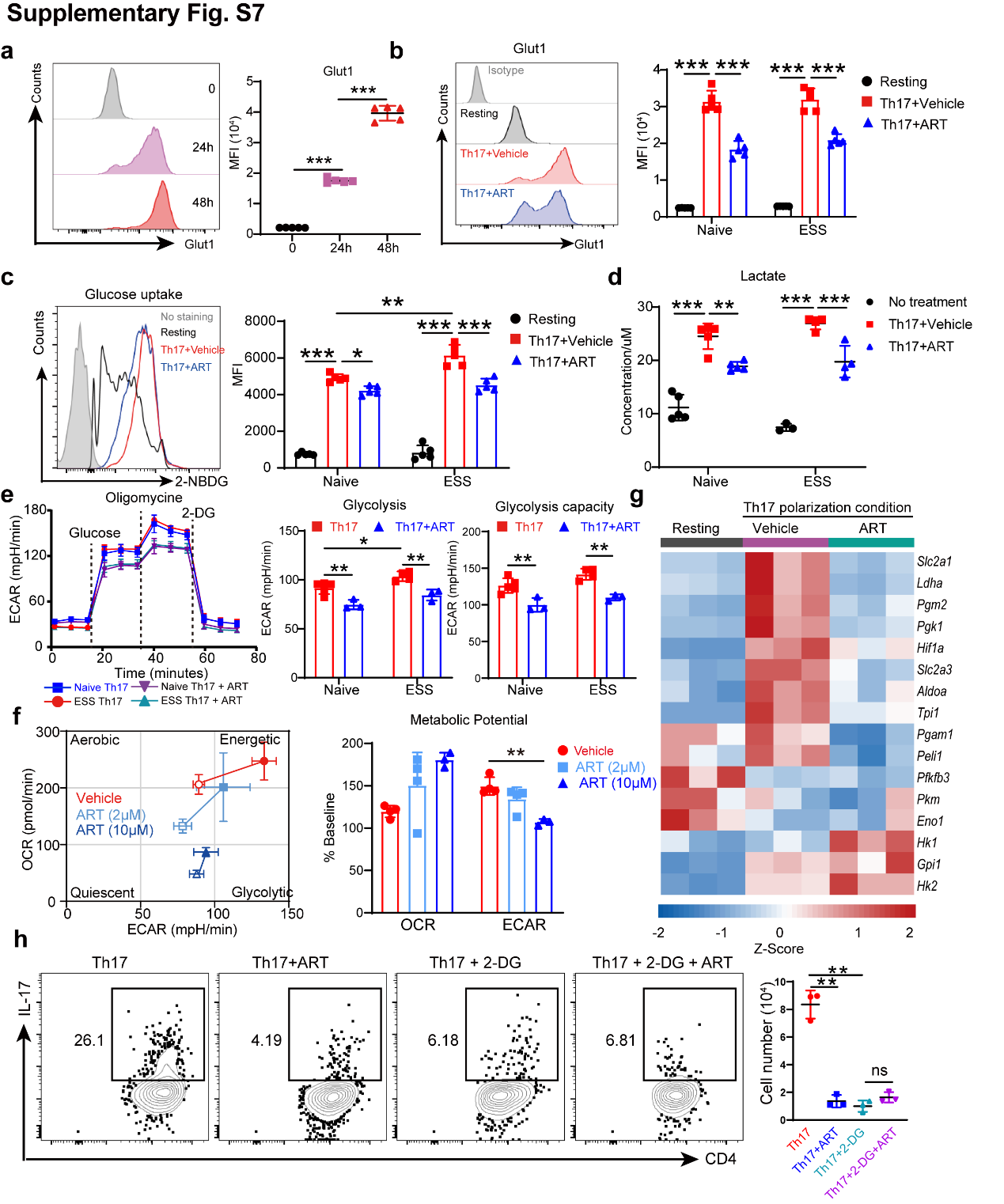


**Supplementary Fig S7.** ART suppresses glycolysis in Th17 cells. **a**, Purified naïve CD4 T cells were cultured under Th17 polarization conditions for 24 and 48 hours. The expression levels of Glut1 were measured by flow cytometry (n=5). **b**, The purified CD4 T cells from normal and ESS mice were cultured under Th17 polarization conditions and treated with vehicle or ART. CD4 T cells without stimulation were collected as resting cells. The expression levels of Glut1 were detected by flow cytometry (n=5). **c**, The cultured Th17 cells with or without ART treatments were incubated with 2-NBDG solution for glucose uptake assay. The fluorescence intensities were analyzed by flow cytometry (n=5). **d**, The lactate concentrations in culture supernatant were measured (n=3). **e**, The cultured Th17 cells from normal and ESS mice were treated with vehicle or ART and were collected for seahorse glycolysis stress test. ECAR values were monitored with sequential addition of glucose, oligomycin, and 2-deoxy-glucose (2-DG). The ECAR values representing glycolysis and glycolysis capacity were calculated and analyzed (n=3~5). **f**, Cultured Th17 cells with or without ART treatments were collected for seahorse cell energy phenotype test. ECAR and OCR values were monitored before (baseline) and after (stressed) addition of oligomycin plus FCCP. Cell energy diagram showing ECAR and OCR at baseline (open symbols) and stressed (closed symbols) conditions was presented. The stressed metabolic potential was analyzed (n=3~4). **g**, Purified naïve CD4 T cells were cultured under Th17 polarization conditions and treated with ART for 48 hours. The relative expression levels of glycolytic genes were examined by Q-PCR analysis. A heatmap showing the expression of indicated genes was presented. **h**, CD4 T cells were cultured under Th17 conditions and treated with ART (2 µM), 2-DG (1 mM), or combinations. Th17 cells were analyzed by flow cytometry (n=3). Data were obtained from at least three independent experiments and presented as mean ± SD; one-way ANOVA; *P < 0.05; **P < 0.01; ***P < 0.001.


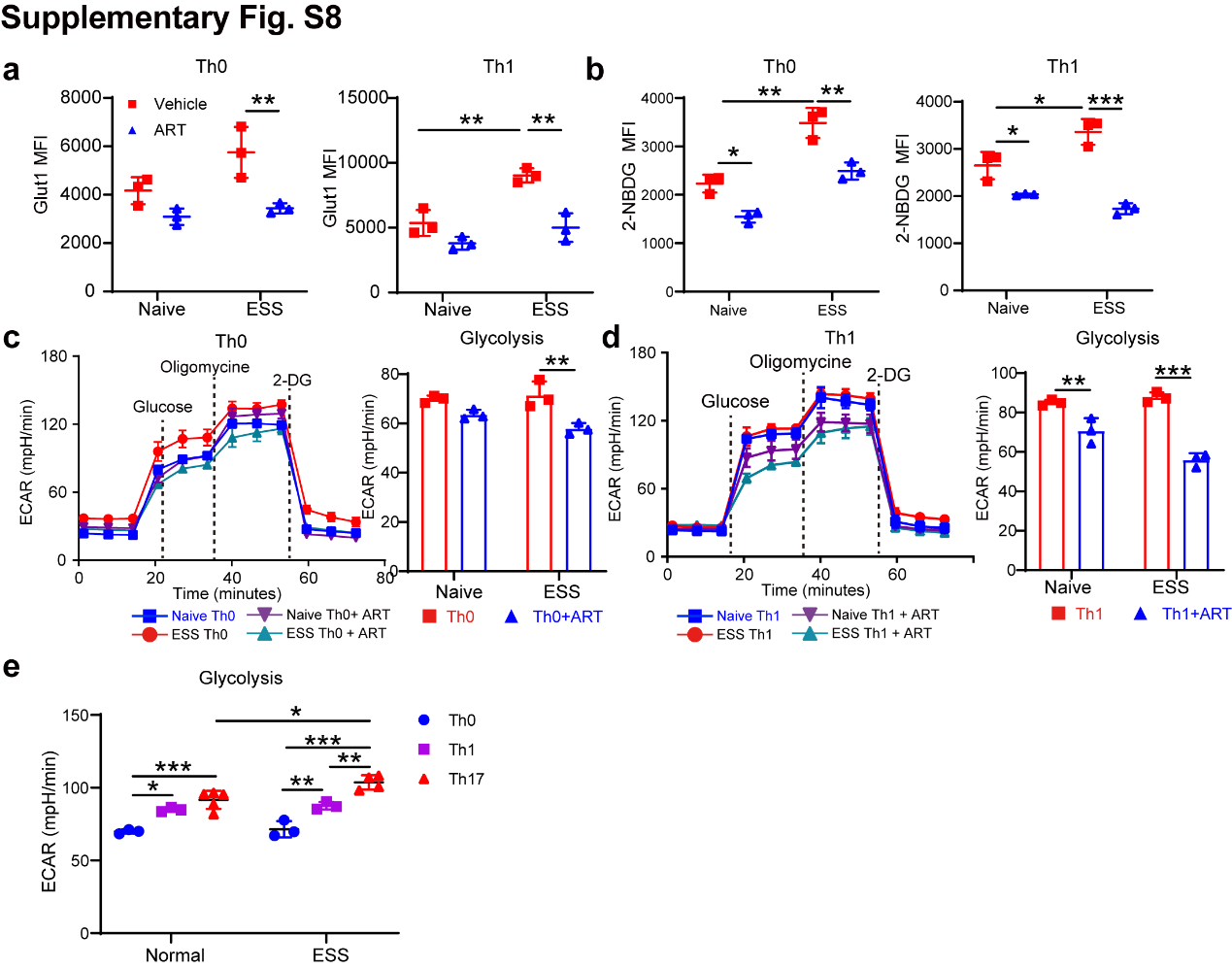


**Supplementary Fig S8.** ART suppresses glycolysis of T cell subsets. **a-d**, CD4 T cells from normal and ESS mice were cultured under Th0 and Th1 conditions and treated with vehicle or ART (2 µM). The expression levels of Glut1 were detected by flow cytometry (a, n=3). The 2-NBDG fluorescence intensities were analyzed by glucose uptake assay (b, n=3). Glycolysis were analyzed by seahorse glycolysis stress test (c-d, n=3). **e**, The glycolysis levels of Th0, Th1 and Th17 cells were analyzed (n=3-5). Data were obtained from at least three independent experiments and presented as mean ± SD; two-way ANOVA; *P < 0.05; **P < 0.01; ***P < 0.001.


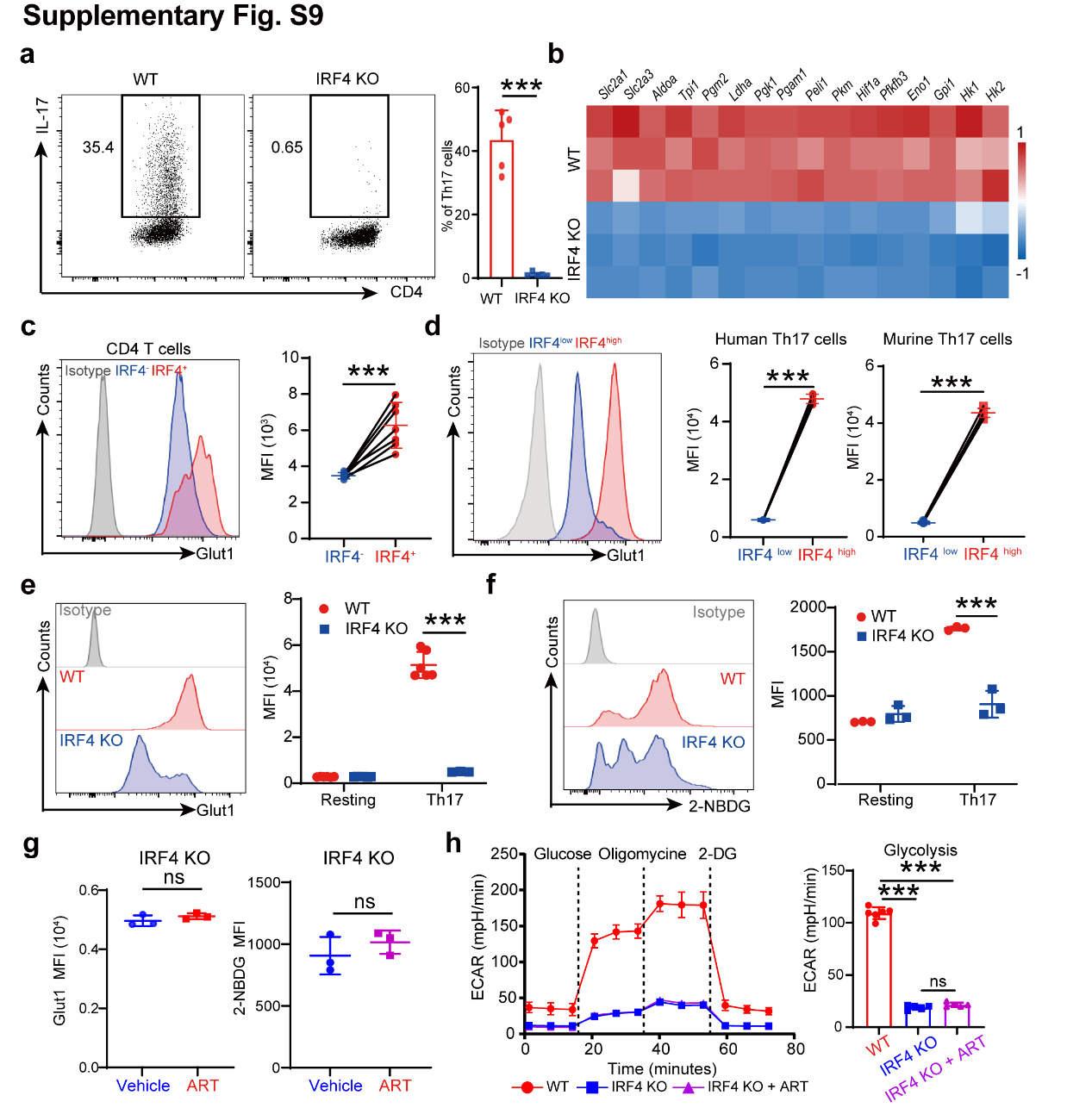


**Supplementary Fig S9.** IRF4 regulates glycolysis in Th17 cells. **a**, Wild-type (WT) and IRF4 knockout (KO) CD4 T cells were cultured under Th17 differentiation conditions. Th17 cells were detected by flow cytometry and the percentages of polarized Th17 cells were analyzed (n=5). **b**, A heatmap plot showing the relative expression levels of glycolytic genes in WT and IRF4 KO CD4 T cells under Th17 differentiation conditions was shown. **c**, The expression levels of Glut1 in IRF4^+^ and IRF4^-^ CD4 T cells from ESS mice were analyzed by flow cytometry (n=7). **d**, The expression levels of Glut1 in IRF4 ^high^ and IRF4 ^low^ polarized human and murine Th17 cells were analyzed (n=6). **e-f**, Glut1 expression levels (**e**) and glucose uptake (**f**) in WT and IRF4 KO CD4 T cells under resting and Th17 polarization conditions were examined (n=6 for **e**, n=3 for **f**). **g**, IRF4 KO CD4 T cells were cultured under Th17 conditions and treated with or without ART (2 µM). Glut1 expression and glucose uptake were analyzed (n=3). h, Glycolysis in WT Th17 cells and IRF4 KO CD4 T cells cultured under Th17 polarization condition with or without ART treatments was determined by seahorse glycolysis stress test (n=5-6). Data were obtained from at least three independent experiments and presented as mean ± SD; unpaired t-test (**a** and **e-g**), paired t-test (**c-d**) and one-way ANOVA (**h**); ns, not significant; *P < 0.05;**P < 0.01; ***P < 0.001.


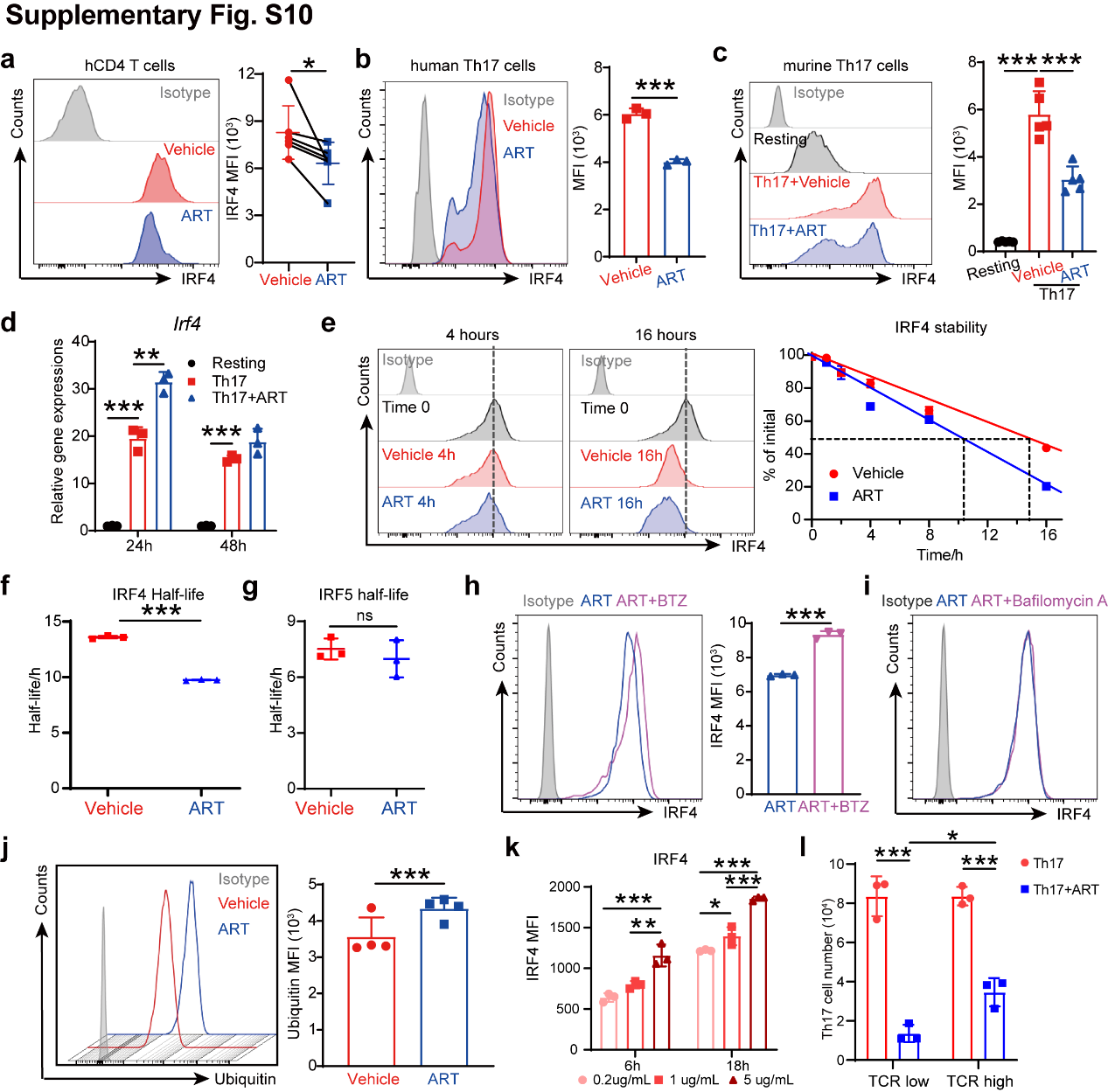


**Supplementary Fig S10.** ART increases proteasomal degradation of IRF4 in Th17 cells. **a**, Expression levels of IRF4 in human CD4 T cells in humanized SS mice with or without ART treatment were detected by flow cytometry (n=6). **b-c**, Purified human (**b**) and murine (**c**) CD4 T cells were cultured under Th17 differentiation condition and treated with ART for 48 hours. The expression levels of IRF4 were detected by flow cytometry (n=3 for human Th17, n=5 for murine Th17). **d**, Purified CD4 T cells were cultured under Th17 differentiation condition and treated with ART. Relative gene expression levels of *Irf4* were measured by Q-PCR analysis (n=3). **e-f**, The cultured Th17 cells were treated with vehicle or ART in the presence of cycloheximide. IRF4 levels were quantified by flow cytometric analysis. Representative histogram and kinetic curves showing IRF4 levels before (time 0) and after ART treatments were shown (**e**). Half-life time was analyzed (**f**, n=3). **g**, The half-life time of IRF5 was determined (n=3). **h-i**, The cultured Th17 cells were treated with Bortezomib (BTZ, **h**) and Bafilomycin A (**i**) in the presence of ART and cycloheximide. The levels of IRF4 were detected by flow cytometry (n=3). **j**, The cultured Th17 cells were treated with vehicle or ART. The linkage-specific K48 ubiquitin levels in IRF4^+^ cells were determined by flow cytometry (n=4). **k**, Naïve CD4 T cells were cultured under Th17 polarization condition with different densities of plate-coated anti-CD3. IRF4 expression levels were determined by flow cytometry (n=3). **l**, CD4 T cells were cultured under Th17 polarization condition with plate-coated anti-CD3 at 1 µg/mL (low) and 5 µg/mL (high). Th17 cells were enumerated by flow cytometry (n=3). Data were obtained from at least three independent experiments and presented as mean ± SD; paired t-test (a), unpaired t-test (b and f-j), one-way ANOVA (c-d, k), two-way ANOVA (l); *P < 0.05; **P < 0.01; ***P < 0.001.

**Supplementary table 1 Primers for Q-PCR analysis**

| **Name** | **Direction** | **Sequence** |
| --- | --- | --- |
| *18s rRNA* | F | AACCCGTTGAACCCCATT |
| *18s rRNA* | R | CCATCCAATCGGTAGTAGCG |
| *Slc2a1* | F | GCTTCTCCAACTGGACCTCAAAC |
| *Slc2a1* | R | ACGAGGAGCACCGTGAAGATGA |
| *Slc2a3* | F | CCGCTTCTCATCTCCATTGTCC |
| *Slc2a3* | R | CCTGCTCCAATCGTGGCATAGA |
| *Hk1* | F | GAAAGGAGACCAACAGCAGAGC |
| *Hk1* | R | TTCGTTCCTCCGAGATCCAAGG |
| *Hk2* | F | CCCTGTGAAGATGTTGCCCACT |
| *Hk2* | R | CCTTCGCTTGCCATTACGCACG |
| *Pfkfb3* | F | TCATCGAGTCGGTCTGTGACGA |
| *Pfkfb3* | R | CATGGCTTCTGCTGAGTTGCAG |
| *Aldoa* | F | CACGAGACACTGTACCAGAAGG |
| *Aldoa* | R | TTGTCTCGCCATTGGTTCCTGC |
| *Tpi1* | F | GGCAACTGGAAGATGAACGGGA |
| *Tpi1* | R | CTGGCAAAGTCGATGTAAGCGG |
| *Gpi1* | F | CCATCAAGGTGGACGGCAAAGA |
| *Gpi1* | R | CCGTGATGGATTTGCCAGTGTAC |
| *Pgam1* | F | CCCCTTCTACAGCAACATCAGC |
| *Pgam1* | R | GCTCTGGCAATAGTGTCCTTCAG |
| *Hif1a* | F | CCTGCACTGAATCAAGAGGTTGC |
| *Hif1a* | R | CCATCAGAAGGACTTGCTGGCT |
| *Pgm2* | F | AGCCAATGACCCAGATGCTGAC |
| *Pgm2* | R | TCCAGGAAGTGAAGAGCCACCA |
| *Eno1* | F | TACCGCCACATTGCTGACTTGG |
| *Eno1* | R | GCTTGTTGCCAGCATGAGAACC |
| *Pgk1* | F | GATGCTTTCCGAGCCTCACTGT |
| *Pgk1* | R | ACCAGCCTTCTGTGGCAGATTC |
| *Pkm* | F | CAGAGAAGGTCTTCCTGGCTCA |
| *Pkm* | R | GCCACATCACTGCCTTCAGCAC |
| *Ldha* | F | ACGCAGACAAGGAGCAGTGGAA |
| *Ldha* | R | ATGCTCTCAGCCAAGTCTGCCA |
| *Peli1* | F | TGTGGGAACGTCTTCAGTCTGC |
| *Peli1* | R | CAGCAAGGTTGCACCACAAAGG |
| *Irf4* | F | GAACGAGGAGAAGAGCGTCTTC |
| *Irf4* | R | GTAGGAGGATCTGGCTTGTCGA |

# Reference:

1. Xiao, F. *et al.* IL-17 drives salivary gland dysfunction via inhibiting TRPC1-mediated calcium movement in Sjögren’s syndrome. *Clin. Transl. Immunol.* **10**, e1277 (2021).

2. Xiao, F. *et al.* Animal models of Sjögren’s syndrome: an update. *Clin. Exp. Rheumatol.* **37 Suppl 118**, 209–216 (2019).

3. Young, N. A. *et al.* A chimeric human–mouse model of Sjögren’s syndrome. *Clin. Immunol.* **156**, 1–8 (2015).

4. Lin, X. *et al.* Th17 cells play a critical role in the development of experimental Sjögren’s syndrome. *Ann. Rheum. Dis.* **17**, 1302–1310 (2015).

5. Scardina, G. A. *et al.* Diagnostic evaluation of serial sections of labial salivary gland biopsies in Sjögren’s syndrome. *Med. Oral Patol. Oral Cirugia Bucal* **12**, E565-568 (2007).

6. Xiao, F. *et al.* Proteasome inhibition suppresses Th17 cell generation and ameliorates autoimmune development in experimental Sjögren’s syndrome. *Cell. Mol. Immunol.* **14**, 924–34 (2017).

7. Hong, X. *et al.* Single-Cell RNA Sequencing Reveals the Expansion of Cytotoxic CD4+ T Lymphocytes and a Landscape of Immune Cells in Primary Sjögren’s Syndrome. *Front. Immunol.* **11**, (2021).
